# Long QT Syndrome and Sinus Bradycardia–A Mini Review

**DOI:** 10.3389/fcvm.2018.00106

**Published:** 2018-08-03

**Authors:** Ronald Wilders, Arie O. Verkerk

**Affiliations:** ^1^Department of Medical Biology, Amsterdam University Medical Centers, Amsterdam, Netherlands; ^2^Department of Experimental Cardiology, Amsterdam University Medical Centers, Amsterdam, Netherlands

**Keywords:** mutations, sinus bradycardia, human, long-QT syndrome, heart rate, sinoatrial node, ion channel, computer simulation

## Abstract

Congenital long-QT syndrome (LQTS) is an inherited cardiac disorder characterized by the prolongation of ventricular repolarization, susceptibility to Torsades de Pointes (TdP), and a risk for sudden death. Various types of congenital LQTS exist, all due to specific defects in ion channel-related genes. Interestingly, almost all of the ion channels affected by the various types of LQTS gene mutations are also expressed in the human sinoatrial node (SAN). It is therefore not surprising that LQTS is frequently associated with a change in basal heart rate (HR). However, current data on how the LQTS-associated ion channel defects result in impaired human SAN pacemaker activity are limited. In this mini-review, we provide an overview of known LQTS mutations with effects on HR and the underlying changes in expression and kinetics of ion channels. Sinus bradycardia has been reported in relation to a large number of LQTS mutations. However, the occurrence of both QT prolongation and sinus bradycardia on a family basis is almost completely limited to LQTS types 3 and 4 (LQT3 and Ankyrin-B syndrome, respectively). Furthermore, a clear causative role of this sinus bradycardia in cardiac events seems reserved to mutations underlying LQT3.

## Introduction

Congenital long-QT syndrome (LQTS) is an inherited cardiac disorder characterized by the prolongation of ventricular repolarization, susceptibility to Torsades de Pointes (TdP), and a risk for sudden death (1). Various types of congenital LQTS exist, but the most common forms of LQTS, accounting for ≈90% of genotype-positive LQTS cases (2), are LQT1, LQT2, and LQT3, caused by mutations in the genes encoding the pore-forming α-subunits of the ion channels carrying the slow delayed rectifier K^+^ current (I_Ks_), rapid delayed rectifier K^+^ current (I_Kr_), and fast Na^+^ current (I_Na_), respectively [for reviews, see (3, 4) and, more recent, (5, 6)]. The incidence and occurrence of phenotype is modulated by a large number of conditional factors (4), including heart rate (HR) (7). For example, LQT1 patients are found to be at greatest risk for cardiac events during conditions of elevated HR, while slower HR provokes cardiac events in LQT2 and LQT3 patients (7). Modulation of HR by exercise may also be a diagnostic criterion in LQTS (8), and treatment/prevention of cardiac events in LQTS is frequently accomplished by HR control (7, 9).

Interestingly, almost all of the ion channels affected by the various types of LQTS gene mutations are also expressed in the human sinoatrial node (SAN) (10). It is therefore not surprising that LQTS is frequently associated with a change in basal HR due to impaired SAN pacemaker activity (11). For example, bradycardia is frequently observed in LQT1 mutation carriers, especially in the fetal-neonatal period (12, 13). It has even been concluded that sinus bradycardia in the cardiotocogram may indicate LQTS in the fetus (14) and that fetal bradycardia is an important predictor of LQTS (15). Also, basal HR was found to be significantly slower in patients with LQT1 compared with non-carriers (16). Maximum HR during exercise may also be reduced in LQTS [see (8, 11), and primary references cited therein]. Thus, LQTS may have a direct impact on HR (17), but this is not a consistent finding (18, 19). One may argue that this is because effects on HR differ between types of LQTS and between specific mutations. However, even within a single mutation different effects on HR are described. For example, the A341V mutation in *KCNQ1* may result in sinus bradycardia (13), but may also occur in absence of baseline HR changes compared to non-carriers (19).

Because LQTS-related rhythm disorders can be triggered by slow or high HR and sinus pauses (4, 11), detailed knowledge of the relation between LQTS and SAN function is required. In this mini-review, we provide an overview of known LQTS mutations with effects on HR and the underlying changes in expression and kinetics of mutant channels.

## LQTS gene mutations and changes in basal heart rate

In Tables S1–S8, which are part of our Supplementary Material, we provide a detailed overview of the various autosomal dominant LQTS mutations known to date that are associated with sinus bradycardia, together with data on the mutation-induced changes in expression and kinetics of the respective ion channels. Below, we provide a brief overview of the various types of congenital LQTS and the extent to which each type is associated with sinus bradycardia. This overview is accompanied by Table 1, which summarizes the data of Tables S1–S8.

**Table 1:** Mutations observed in patients with both sinus bradycardia and LQTS^a^.

**LQTS type**	**Gene**	**Protein**	**Patient groups**	**Mutations**
LQT1	*KCNQ1*	K_V_7.1	Single patient	c.387-5 T>A, R174H, L175fsX, G179S, G325R, S338F, F339S, F339del, A344V, K422fsX, T587M, A590T
			Multiple single patients	R231C, A341V, D611Y
LQT2	*KCNH2*	K_V_11.1	Single patient	R534C, A561V
			Small family	K638del
LQT3	*SCN5A*	Na_V_1.5	Small family	QKP1507–1509del
			Large family	1795insD
			Multiple families	KPQ1505–1507del (ΔKPQ), E1784K
LQT4	*ANK2*	Ankyrin-B	Single patient	I1855R
			Multiple single patients	R1788W
			Multiple families	E1425G
LQT5	*KCNE1*	KCNE1 (minK)	Single patient	A8V, D85N, R98W
			Multiple single patients	D85N
			Small family	D85N
LQT6	*KCNE2*	KCNE2 (MirP1)	Multiple single patients	M54T
LQT7	*KCNJ2*	Kir2.1	–	–
LQT8	*CACNA1C*	Ca_V_1.2	Single patient	A582D, P857R, R858H
LQT9	*CAV3*	Caveolin-3	Multiple single patients	T78M
LQT10	*SCN4B*	Na_V_β4	Single patient	L179F
LQT11	*AKAP9*	Yotiao	–	–
LQT12	*SNTA1*	α1-syntrophin	–	–
LQT13	*KCNJ5*	Kir3.4 (GIRK4)	–	–
LQT14	*CALM1*	Calmodulin	Single patient	E105A
			Multiple single patients	F142L
LQT15	*CALM2*	Calmodulin	Single patient	D96V, N98I, D132H
LQT16	*CALM3*	Calmodulin	Single patient	D96H, F142L

a *Further details are provided in Tables S1–S8, which are part of our Supplementary Material*.

### LQT1

LQT1 is due to loss-of-function mutations in *KCNQ1*, the gene encoding the pore-forming α-subunit of the I_Ks_ channel (K_V_7.1). A decrease in I_Ks_ will result in a prolongation of the ventricular action potential (AP) and a prolongation of the QT interval on the ECG (20). Of note, four K_V_7.1 α-subunits assemble in a tetramer to create the pore of an I_Ks_ channel. Therefore, a mutation in *KCNQ1* may affect a large majority of the I_Ks_ channels as wild-type and mutant subunits co-assemble in heterotetramers.

Many *KCNQ1* mutations exist and some are associated with sinus bradycardia (Table 1). These bradycardia-associated mutations result in “loss-of-function” by a reduced level of channel expression, expression of non-functional channels, activation at more positive membrane potentials, faster deactivation kinetics, and/or inhibited cAMP-dependent stimulation. For example, the A341V mutation strongly suppresses the increase in I_Ks_ in response to cAMP (21), which may also explain the more pronounced phenotype during exercise. Sinus bradycardia in LQT1 patients seems limited to isolated, often neonate cases (Table S1).

It is somewhat difficult to envision how a loss of repolarizing I_Ks_ *per se* would lead to a profound increase in the cycle length of SAN cells, thus generating sinus bradycardia. Such loss would lengthen AP duration (APD), but at the same time shorten the considerably longer (22) diastolic phase by increasing the rate of diastolic depolarization. An increase in repolarizing I_Ks_, on the other hand, as observed in short QT syndrome type 2 (SQT2), will inhibit diastolic depolarization and substantially increase cycle length, despite an accompanying decrease in APD, as observed in simulations by Fabbri et al. (23) using their recently developed comprehensive computer model of a single human SAN pacemaker cell. Clinically, sinus bradycardia is indeed relatively common in SQT2 patients [see, e.g., (24)].

### LQT2

LQT2 is due to loss-of-function mutations in *KCNH2*, the gene encoding the pore-forming α-subunit of the I_Kr_ channel (K_V_11.1). Observation of sinus bradycardia in LQT2 patients seems rare (25, 26) and limited to a few isolated cases and a small family (Table 1). Bradycardia does occur in the fetal-neonatal period, but is due to 2:1 atrioventricular block rather than sinus bradycardia (12). Such cases are not included in Table 1. In contrast, Horigome et al. (13) reported that the incidence of sinus bradycardia was comparable between groups of young (<1 year, mostly fetal-neonatal) LQT1, LQT2, and LQT3 patients. However, whether the LQTS observation is due to the bradycardia or the bradycardia results from the mutations (4, 11) is less clear. Bradycardia-associated mutations in *KCNH2*, so far characterized, result in a decrease in current density, non-functional channels, a shift in voltage of half-activation, and faster deactivation and inactivation rates (Table S2).

The above consideration regarding the potential association between sinus bradycardia and the increase in repolarizing I_Ks_ in case of SQT2 similarly holds for the increase in repolarizing I_Kr_ in case of short QT syndrome type 1 (SQT1). Sinus bradycardia is indeed observed in SQT1 patients, although being less common than in SQT2 patients (27).

### LQT3

LQT3 is due to gain-of-function mutations in *SCN5A*, the gene encoding the pore-forming α-subunit of the I_Na_ channel (Na_V_1.5). Unlike LQT1 and LQT2, the occurrence of sinus bradycardia is not limited to isolated cases. Several families, including the large Dutch family with the 1795insD founder mutation (28), show both QT prolongation and sinus bradycardia (Table 1). A common feature is the increased late current, also named persistent or sustained current, underlying the QT prolongation. Another common feature is the decrease in “window current” due to a positive shift in the steady-state activation curve and/or a negative shift in the steady-state inactivation curve (Table S3).

**Figure 1:**
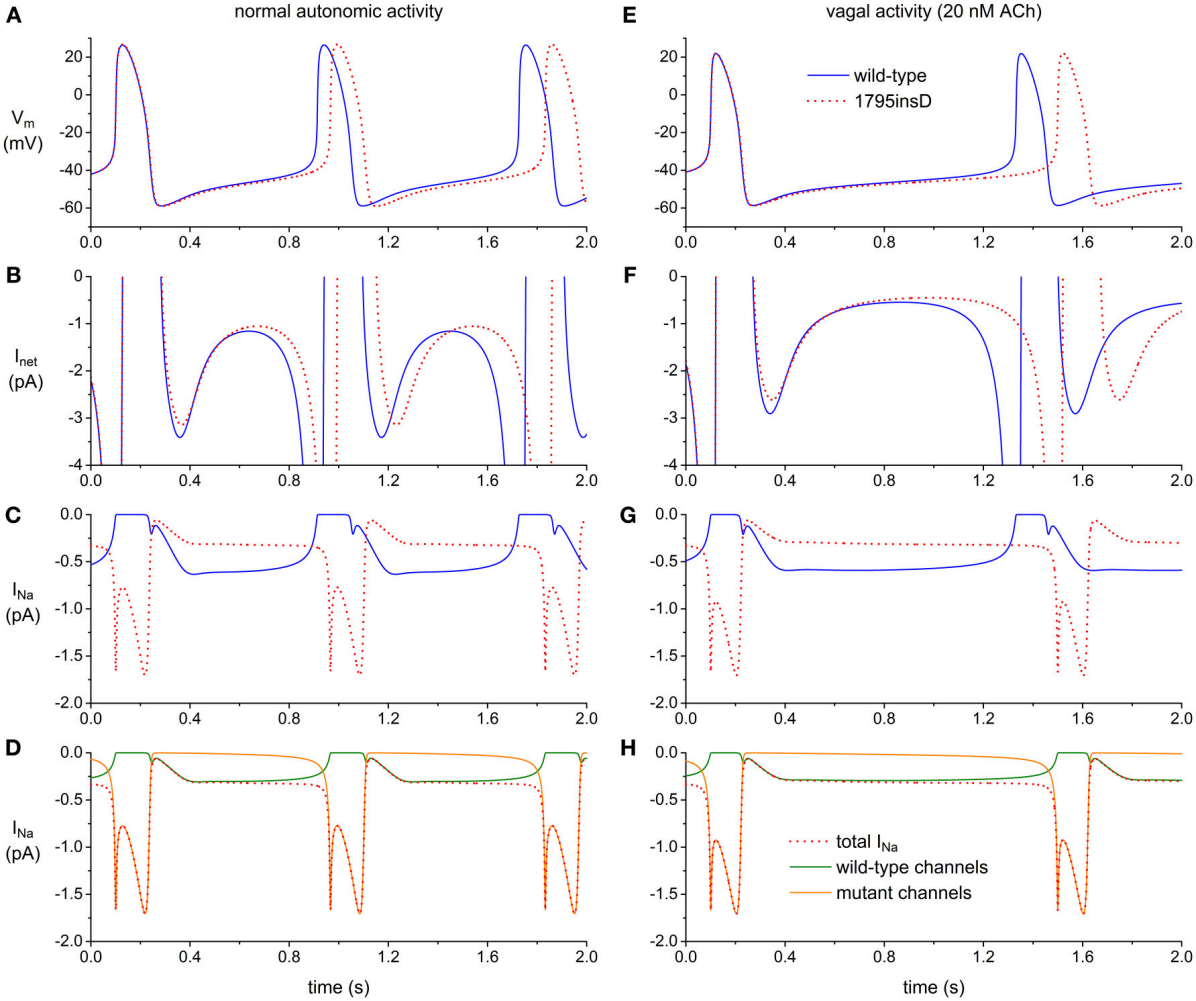
Effect of the 1795insD mutation in *SCN5A* on the electrical activity of the Fabbri–Severi model cell of a human SAN pacemaker cell **(A–D)** under control conditions (normal autonomic activity) and **(E–H)** during vagal activity [based on data from recent computer simulations (29)]. **(A,E)** Membrane potential (V_m_) of wild-type and mutant cell (solid blue and dotted red trace, respectively). **(B,F)** Associated net membrane current (I_net_). (**C,G**) Associated fast Na^+^ current (I_Na_). **(D,H)** Contribution of wild-type and mutant channels (solid green and orange trace, respectively) to I_Na_ of the mutant cell (dotted red trace).

Figures 1A–D illustrate the effects of the 1795insD mutation, based on data from recent computer simulations (29), as set out in the Supplementary Material. The observed increase in cycle length (Figure 1A) is largely due to a decrease in net inward current (I_net_) during diastole (Figure 1B), which in turn is due to a striking change in the time course of I_Na_ (Figure 1C). Where I_Ks_ and I_Kr_ channels are tetramers, the pore of the I_Na_ channel is formed by a single Na_V_1.5 protein. As a consequence, “mutant I_Na_” (Figure 1D, dotted red trace) is partly flowing through pure wild-type channels (solid green trace) and partly through pure mutant channels (solid orange trace). There is hardly any current flowing through these mutant channels during diastole due to the decrease in window current. There is, on the other hand, some late current flowing during the AP, albeit with a negligible effect on APD, in contrast to ventricular myocytes, in which the current density of I_Na_ is much larger. These effects are more pronounced during vagal activity (Figures 1E–H). The slight decrease in diastolic depolarization rate and increase in cycle length as a result of the inhibition of I_Na_ (Figures 1A,E) are in line with experimental observations on isolated rabbit SAN cells (30).

The increase in late current (“gain-of-function”) is a prerequisite for QT prolongation, but not for sinus bradycardia. A sole decrease in window current (“loss-of-function”), as for example observed in case of the R376C and D1275N mutations in *SCN5A* (31, 32), is sufficient to cause sinus bradycardia.

### LQT4

Ankyrin-B syndrome, originally named LQT4, is due to heterozygous loss-of-function mutations in *ANK2*, encoding the widely distributed ankyrin-B adaptor protein. Loss of ankyrin-B results in Ca^2+^ homeostasis dysfunction by reduced Na^+^-Ca^2+^ exchange current (I_NCX_), L-type Ca^2+^ current (I_Ca,L_), Na^+^-K^+^-ATPase, and IP3 receptor expression (Table S4). Mutations in *ANK2* associated with both QTc prolongation and sinus bradycardia are observed in both large families and single patients (Table 1).

### LQT5

LQT5 is due to loss-of-function mutations in *KCNE1*. The encoded protein, named KCNE1 or minK, is a β-subunit that may affect both I_Ks_ and I_Kr_ function. Reports of bradycardia in LQT5 patients are scarce (Table 1). The observations made to date show reduced I_Ks_ or I_Kr_ density or a shift of I_Ks_ activation to more positive potentials (Table S5). Interestingly, the A8V mutation affects I_Ks_ but not I_Kr_, whereas the R98W mutation affects I_Kr_ but not I_Ks_.

### LQT6

LQT6 is due to loss-of-function mutations in *KCNE2*. The encoded protein, named KCNE2 or MirP1, is a β-subunit that may affect various ion currents. Mutations in *KCNE2* may result in an accelerated inactivation time course of I_Kr_ (33, 34), but also in an increase of I_Ca,L_ (35), and a reduction of the hyperpolarization-activated current (I_f_) (36), the latter important for pacemaker activity in human SAN cells (22). Despite its multiple ion current modulations, *KCNE2* mutations associated with sinus bradycardia are limited to M54T and V65M. In case of the M54T mutation, both I_Kr_ and I_f_ are inhibited (Table S6). It is conceivable that the V65M mutation also acts through I_f_, given the well-established effect of KCNE2 on I_f_ (37).

### LQT7

Andersen-Tawil syndrome (“LQT7”) is a multisystem disorder due to loss-of-function mutations in *KCNJ2*, the gene encoding the Kir2.1 protein, which assembles in tetramers to build the channels that carry the inward rectifier K^+^ current (I_K1_) (38). Given the low expression of Kir2.1 in human SAN (10), it is not surprising that HR seems not affected in Andersen-Tawil syndrome patients (39).

### LQT8

Timothy syndrome (TS) is a severe multisystem disorder due to gain-of-function mutations in *CACNA1C*, encoding the pore-forming α-subunit of the I_Ca,L_ channel (Ca_V_1.2), and results in bradycardia in almost all patients known, but caused by 2:1 atrioventricular block rather than sinus bradycardia (see footnote to Table S7). Although TS is also known as LQT8, because of the extreme QT prolongation in TS patients (40, 41), we restricted the LQT8 data in Table S7 to non-TS patients. In isolated cases, these show sinus bradycardia (Table 1). Mutant I_Ca,L_ shows an increase in density or slowing of inactivation (Table S7).

### LQT9

LQT9 is due to mutations in *CAV3*, encoding caveolin-3, an important structural component of caveolae membrane in muscle cells (42). *CAV3* mutations in heart have been shown to increase the late I_Na_, thus causing QT prolongation as in LQT3 (43, 44). More recently, it has been shown that mutations in *CAV3* may affect several other membrane currents (see footnote to Table S8). Sinus bradycardia has been observed in two patients carrying the T78M mutation (Table 1).

### LQT10

LQT10 is due to gain-of-function mutations in *SCN4B*, encoding the Na_V_β4 β-subunit of the I_Na_ channel. A case report exists for an *SCN4B*-L179F mutation with impact on SAN function (Table 1). In a 21-month-old girl, profound QT prolongation and bradycardia (<60 bpm) were observed (45). The *SCN4B*-L179F mutation increases late I_Na_ (Table S8) and may thus have effects comparable to LQT3 mutations.

### LQT11–LQT16 and beyond

To the best of our knowledge, no sinus bradycardia has been reported in relation to the rare LQTS types LQT11–LQT13 (see footnote to Table S8). Genetic variation in *KCNJ3* and *KCNJ5*, encoding the pore-forming Kir3.1 and Kir3.4 ion channel subunits of the acetylcholine-sensitive K^+^ current (I_K,ACh_), and which the latter may underlie LQT13, seems not involved in pathogenesis of SAN dysfunction (46). However, it is suggested that identification of susceptibility genes for SAN dysfunction requires the construction of a large database of patients and controls whose phenotype should be identified with standard criteria to ensure adequate power for cause-effect studies (47). Thus, the incidence of some LQTS types may be too low to determine clear associations with bradycardia.

Several reports exist of mutations in the *CALM1–CALM3* genes, each encoding the ubiquitous Ca^2+^ sensing protein calmodulin, in relation to LQTS and sinus bradycardia (Table 1 and Table S8). Calmodulin regulates multiple Ca^2+^-related processes in the cardiomyocyte (48), including, e.g., gating of the I_Ks_ channel (49). Mutations in *CALM1* and *CALM2* may impair Ca^2+^-dependent inactivation of I_Ca,L_ (50, 51), functionally comparable to the slowed inactivation of I_Ca,L_ in case of LQT8 (Table S7).

In Table 1 and Table S8, we, like others (52, 53), used LQT14–LQT16 in relation to mutations in *CALM1–CALM3*. We are, however, well aware that the naming LQT16 has been used in other review articles (54, 55) in relation to mutations in *SCN1B* (56) and in *TRDN* (57). Altmann et al. identified autosomal recessive homozygous or compound heterozygous mutations in *TRDN*, encoding triadin, associated with LQTS, and themselves proposed that “triadin knockout syndrome” or “*TRDN*-mediated autosomal-recessive LQTS” should be used rather than “LQT17,” because of the atypical phenotype that was observed (57).

## Discussion and conclusion

SAN action potentials are generated from a delicate balance of several inwardly and outwardly directed ionic currents, and “Ca^2+^ clock” mechanisms [for reviews, see (58–60)]. While LQTS gene mutations may affect HR by changes in SAN action potential repolarization, it is highly likely that they also affect the intrinsic SAN cycle length by changes in the diastolic, phase 4, depolarization rate, as illustrated by the computer simulations of Figure 1.

It is important to realize that a mutation in a single LQTS-related gene may affect several ion currents. This does not only hold for mutations in a Ca^2+^ sensing protein like calmodulin, but also for mutations in the α-subunit of a specific ion channel. The LQT1-related T587M mutation in *KCNQ1* for example does not only reduce I_Ks_, but also fails to increase membrane localization of the *KCNH2*-encoded K_V_11.1 protein, as opposed to wild-type *KCNQ1*, thus also reducing I_Kr_ (61). Furthermore, we have to keep in mind that the LQTS-induced changes in rhythm may in turn induce changes in expression of specific ion channels, as demonstrated in studies by Tsuji et al. (62), Yeh et al. (63), and D'Souza et al. (64).

LQT2 and LQT3, but not LQT1, patients have a more pronounced risk for arrhythmias at slower HR (7). LQT2 and LQT3 gene mutations may therefore increase the risk for cardiac events via a direct effect on HR, as indeed clinically was found in a large family with LQT3 (65, 66). In LQT1 patients, on the other hand, cardiac events tend to occur during exercise (7). These differences between LQTS types 1–3 underscore the differences in underlying mechanisms and the potential role of sinus bradycardia in cardiac events.

As shown in Tables S1–S8 and summarized in Table 1, sinus bradycardia has been reported in relation to a large number of LQTS mutations. However, observations are limited to one or a few single patients for most of these mutations (Table 1). The occurrence of both QT prolongation and sinus bradycardia on a family basis is almost completely limited to LQT3 and Ankyrin-B syndrome (“LQT4”). However, the mechanisms of the associated ventricular arrhythmias and sudden death are largely different. Cardiac events, including nocturnal sudden death, are provoked by the bradycardia and associated excessive QT prolongation in case of LQT3 (65, 66), whereas disturbed calcium homeostasis leads to dysfunction of the SAN cells in case of the Ankyrin-B syndrome, with sudden death occurring after physical exertion and emotional stress (67–70).

We conclude that, although sinus bradycardia has been reported in relation to a large number of LQTS mutations, a causative role of this sinus bradycardia in cardiac events is limited to mutations underlying LQTS type 3.

## Author contributions

RW and AV: experimental design, data acquisition, analysis and interpretation of data, drafting manuscript, editing manuscript, and approval.

### Conflict of interest statement

The authors declare that the research was conducted in the absence of any commercial or financial relationships that could be construed as a potential conflict of interest.
