# Supplementary material for: Long QT Syndrome and Sinus Bradycardia–A Mini Review
Source: Front Cardiovasc Med. 2018 Aug 3;5:106. doi: 10.3389/fcvm.2018.00106 (PMC6085426; doi:10.3389/fcvm.2018.00106)
Supplement: Supplementary file 1 [file Data_Sheet_1.PDF]

Supplementary Material

Long QT Syndrome and Sinus Bradycardia – A Mini Review

Ronald Wilders, Arie O. Verkerk*

*** Correspondence:** Arie O. Verkerk: a.o.verkerk@amc.uva.nl

1 LQTS Mutations Associated with Sinus Bradycardia

Tables S1–S8 below provide a detailed overview of the various congenital long-QT syndrome (LQTS) mutations known to date that are associated with sinus bradycardia, together with data on the mutation-induced changes in expression and kinetics of the respective ion channels. Not included are mutations associated with (often fetal or neonatal) bradycardia as a result of 2:1 or more advanced atrioventricular block, as for example observed by Piippo et al. (2000), Lupoglazoff et al. (2004), and Horigome et al. (2010). Also not included are data on autosomal recessive variants of congenital LQTS that are caused by homozygous or compound heterozygous mutations, like the Jervell and Lange-Nielsen syndrome (Schwartz et al., 2006).

(Continued on next page)

Table S1. Mutations in *KCNQ1* underlying both sinus bradycardia and LQTS type 1 (LQT1)

Mutation	Clinical observations	Functional effects	Source
c.387-5 T>A	Sinus bradycardia in 5 out of 10 non-symptomatic heterozygous mutation carriers (family members of patients with familial autosomal recessive LQTS); QTc prolongation in one these 5 carriers	—	Bdier et al. (2017)
c.387-5 T>A	—	HEK-293 cells co-expressing wild-type and mutant subunits show I_{Ks} similar to wild-type	Bhuiyan et al. (2008)
R174H	Neonatal bradycardia attributable to sinus bradycardia in single male patient; QTc of 500 ms; asymptomatic at 7 y	—	Lupoglazoff et al. (2004)
R174H	—	Strongly reduced channel expression in HEK-293 cells	Huang et al. (2018)
L175fsX (c.524_534dup)	Neonatal bradycardia attributable to sinus bradycardia in single male patient; QTc of 560 ms; asymptomatic at 3 y	Nonsense mutation; non-functional protein	Lupoglazoff et al. (2004)
G179S	Sinus bradycardia and QTc prolongation in one of two non-symptomatic heterozygous mutation carriers (family members of patients with familial autosomal recessive LQTS)	—	Bdier et al. (2017)

(Continued)

Mutation	Clinical observations	Functional effects	Source
G179S	—	Strong decrease in current density, as compared to wild-type, when co-expressed in CHO cells with wild-type *KCNE1*	Vanoye et al. (2017)
R231C	Fetal bradycardia, neonatal sinus bradycardia and QTc prolongation in three male patients; asymptomatic at 2 months, 2 y, and 8 y	—	Lupoglazoff et al. (2004)
R231C	Overlap syndrome with neonatal sinus bradycardia, AF, and LQTS in two families; no patients with both sinus bradycardia and LQTS	Faster activation and deactivation of homomeric *KCNQ1* channels in *Xenopus* oocytes; higher potentials required to activate the channels; altered interactions with KCNE β-subunits	Henrion et al. (2012)
R231C	Neonatal sinus bradycardia in a single male patient; QTc intervals on subsequent ECGs ranging between 392 and 439 ms; AF, but not sinus bradycardia, in two genotype-positive family members	—	Guerrier et al. (2013)
R231C	—	Significant decrease in current density, as compared to wild-type, when co-expressed in CHO cells with wild-type *KCNE1*	Vanoye et al. (2017)
R231C	—	Strongly reduced channel expression in HEK-293 cells	Huang et al. (2018)

(Continued)

Mutation	Clinical observations	Functional effects	Source
G325R	Fetal bradycardia, neonatal sinus bradycardia and QTc prolongation in single female patient; asymptomatic at 2 y	—	Lupoglazoff et al. (2004)
G325R	LQTS and/or syncope in several of 22 mutation carriers from 11 families	Heterozygous *KCNQ1* channels in HEK-293 cells show ≈70% reduction in current amplitude with only small changes in activation gating	Burgess et al. (2012)
G325R	LQTS in single patient	Heterozygous *KCNQ1* channels in *Xenopus* oocytes show >50% reduction in current amplitude and +12-mV shift in voltage dependence of activation	Aidery et al. (2012)
S338F	Sinus bradycardia and profound QTc prolongation in 7-year-old male; also carrier of the D85N variant in *KCNE1*	Dominant-negative effect on function of channels expressed in *Xenopus* oocytes	Hoosien et al. (2013)
F339S	Fetal bradycardia and prolonged QT interval during the first month of life in a single male patient	Dominant-negative effect on function of channels expressed in *Xenopus* oocytes	Hoosien et al. (2013)
F339del	Sinus bradycardia and severe QTc prolongation in 26-year-old female patient; regular heart rates in three family members carrying the mutation	Heterozygous *KCNQ1* channels in *Xenopus* oocytes show >90% reduction in current amplitude	Thomas et al. (2005)

(Continued)

Mutation	Clinical observations	Functional effects	Source
A341V	Fetal or neonatal sinus bradycardia and QTc prolongation in two male patients	–	Hamada et al. (1999); Horigome et al. (2010)
A341V	Baseline HR not different between mutation carriers and non-carriers	Heterozygous *KCNQ1* channels in CHO cells show ≈50% reduction in current amplitude	Brink et al. (2005)
A341V	—	Dominant-negative suppression of cAMP-dependent I_{Ks} upregulation on top of a dominant-negative reduction in basal current in CHO cells	Heijman et al. (2012)
A344V	Sinus bradycardia and QTc prolongation in one of two non-symptomatic heterozygous mutation carriers (family members of patients with familial autosomal recessive LQTS)	—	Bdier et al. (2017)
A344V	—	*Xenopus* oocytes co-expressing wild-type and mutant subunits show +15-mV shift in voltage dependence of I_{Ks} activation	Siebrands et al. (2006)
A344V	—	Strong decrease in current density, as compared to wild-type, when co-expressed in CHO cells with wild-type *KCNE1*	Vanoye et al. (2017)

(Continued)

Mutation	Clinical observations	Functional effects	Source
K422fsX (c.1258insA)	Fetal bradycardia, neonatal sinus bradycardia and mild QTc prolongation in single female patient; asymptomatic at 4 y	Nonsense mutation; non-functional protein	Lupoglazoff et al. (2004)
T587M	Fetal bradycardia, neonatal sinus bradycardia, and QTc prolongation in male neonate	—	Horigome et al. (2010)
T587M	LQTS and syncope at 9 y in three non-familial patients	No dominant-negative suppression of I_{Ks} in COS-7 cells; trafficking deficiency of T587M protein	Yamashita et al. (2001)
T587M	—	Haploinsufficiency observed in CHO cells, reducing I_{Ks} by ≈50%; as opposed to wild-type, T587M fails to increase membrane localization of the *KCNH2*-encoded Kv11.1 protein, thus not only reducing I_{Ks}, but also I_{Kr}	Biliczki et al. (2009)
A590T	Neonatal bradycardia and mild QTc prolongation in single male patient; asymptomatic at 6 y	—	Lupoglazoff et al. (2004)
A590T	—	Heterozygous *KCNQ1* channels in HEK-293 cells show I_{Ks} similar to wild-type	Kinoshita et al. (2014)

(Continued)

Mutation	Clinical observations	Functional effects	Source
D611Y	Sinus bradycardia and QTc prolongation in male neonate; sinus bradycardia in female neonate, but no data on QTc	—	Horigome et al. (2010)
D611Y	Marked QTc prolongation in six out of 11 mutation carriers; one, a 2-year-old boy, diagnosed at birth with bradycardia	Mutant *KCNQ1* channels in *Xenopus* oocytes show increased time constants of current activation compared to wild-type; no difference in current amplitude	Yamaguchi et al. (2005)

Table S2. Mutations in *KCNH2* underlying both sinus bradycardia and LQTS type 2 (LQT2)

Mutation	Clinical observations	Functional effects	Source
R534C	Sinus bradycardia (48 bpm) and marked QT prolongation (QTc 477 ms) in a 60-year-old male with a long history of epilepsy; same mutation and prolonged QTc interval subsequently identified in 57-year-old niece	—	Omichi et al. (2010)
R534C	QT prolongation in a 19-year-old male (QTc 550 ms); QT prolongation in four genotype-positive family members (QTc ranging from 460 to 680 ms)	Heterozygous expression in *Xenopus* oocytes revealed a −9-mV shift in voltage of half-activation, accelerated activation and deactivation, and reduced steady-state inactivation; no suppression of current amplitude	Nakajima et al. (1999)
A561V	Sinus bradycardia and severe QT prolongation (QTc 637 ms) in a 4-day-old girl	—	Beery et al. (2007)
A561V	—	Dominant-negative current suppression in HEK-293 or COS-7 cells due to retention of A561V in the endoplasmic reticulum and tagging wild-type channels for retention by co-assembly with A561V subunits	Ficker et al. (2000)

(Continued)

Mutation	Clinical observations	Functional effects	Source
A561V	—	Co-expression of wild-type and mutant HERG in CHO cells reveals an ≈85% reduction in current amplitude and a −12-mV shift in voltage of half-activation; reduced abundance of co-expressed wild-type HERG in cultured mammalian cells, both by decreasing synthesis of full-length protein and by increasing turnover	Kagan et al. (2000)
A561V	—	Co-expression of wild-type and mutant HERG in COS-7 cells reveals an ≈70% reduction in current amplitude, related to impaired trafficking	Bellocq et al. (2000)
A561V	13-year-old male with a QTc of 584 ms	Amplitude of peak and tail I_{Kr} depressed by 80–90% in human induced pluripotent stem cell-derived cardiomyocytes	Mehta et al. (2014)
A561V	18-year-old male with QTc prolongation and frequent episodes of syncope during physical or emotional stress	Co-expression of wild-type and mutant HERG in HEK-293 cells reveals a strong reduction in current amplitude as well as alterations in activation and inactivation	Li et al. (2016)
A561V	—	Co-expression of wild-type and mutant HERG in HEK-293 cells reveals a 76% reduction in current amplitude	Wang et al. (2018)

(Continued)

Mutation	Clinical observations	Functional effects	Source
K638del	Marked QT prolongation (QTc 597 ms) and sinus bradycardia (51 bpm) in 56-year-old male; also marked QT prolongation and sinus bradycardia in proband's genotype-positive daughter and granddaughter	—	Ichikawa et al. (2016a)

Table S3. Mutations in *SCN5A* underlying both sinus bradycardia and LQTS type 3 (LQT3)

Mutation	Clinical observations	Functional effects	Source
KPQ1505–1507del (ΔKPQ)	Marked QTc prolongation and a history of syncope or aborted sudden death in two unrelated families	—	Wang et al. (1995)
KPQ1505–1507del (ΔKPQ)	Marked QTc prolongation and significant prolongation of RR interval in mutation carriers as compared to unaffected family members in two unrelated families	—	Moss et al. (1995)
KPQ1505–1507del (ΔKPQ)	—	Faster inactivation of ΔKPQ channels expressed in *Xenopus* oocytes; no change in recovery from inactivation; −6-mV shift in voltage of half-inactivation; substantial persistent current	Bennett et al. (1995)
KPQ1505–1507del (ΔKPQ)	—	ΔKPQ channels expressed in *Xenopus* oocytes reveal a persistent current at −10 mV of ≈1.7% of peak I_{Na}, as compared to ≈0.15% for wild-type	Dumaine et al. (1996)
KPQ1505–1507del (ΔKPQ)	—	Faster inactivation and recovery from inactivation of ΔKPQ channels expressed in HEK-293/tsA201 cells; persistent current at −20 mV of 2.57 ± 0.27% of peak I_{Na}; +6-mV shift in voltage of half-activation	Wang et al. (1996)

(Continued)

Mutation	Clinical observations	Functional effects	Source
KPQ1505–1507del (ΔKPQ)	—	Faster inactivation and recovery from inactivation of ΔKPQ channels expressed in HEK-293 cells; no shifts in voltage of half-activation and voltage of half-inactivation; substantial persistent current	Chandra et al. (1998)
KPQ1505–1507del (ΔKPQ)	Slowed atrial and ventricular conduction in mutation carriers	—	Zareba et al. (2001)
KPQ1505–1507del (ΔKPQ)	Bradycardia and QT prolongation in heterozygous mice carrying the ΔKPQ mutation	Two-fold increase in I_{Na} density, as compared to wild-type, in ventricular myocytes isolated from the heterozygous ΔKPQ mice; large, slowly inactivating persistent I_{Na}; no differences in steady-state activation and inactivation	Nuyens et al. (2001)
KPQ1505–1507del (ΔKPQ)	Resting heart rate of 57 bpm and QTc interval of 534 ms in six male mutation carriers	—	Moss et al. (2005)
KPQ1505–1507del (ΔKPQ)	—	ΔKPQ channels expressed in HEK-293 cells reveal a +20-mV shift in voltage of half-activation and a −10-mV shift in voltage of half-inactivation as compared to wild-type	Spencer (2009)

(Continued)

Mutation	Clinical observations	Functional effects	Source
QKP1507–1509del	41-year-old woman diagnosed with LQT3, RR interval of 1240 ms and QTc of 576 ms; 12-year-old genotype-positive asymptomatic son with RR interval of 1250 ms and QTc of 595 ms	Na^+ channels expressed in tsA201 cells reveal a +12-mV shift in voltage of half-activation, a slightly fastened recovery from inactivation and a persistent I_{Na} of ≈2% of peak I_{Na} at −30 mV	Keller et al. (2003)
E1784K	Sudden death at rest in 13-year-old girl carrying the mutation; QT prolongation and sinus bradycardia in four genotype-positive family members	Na^+ channels expressed in *Xenopus* oocytes reveal a −12-mV shift in steady-state inactivation curve as compared to wild type; currents of similar magnitude, but 2– 4% residual current in E1784K and no residual current in wild type	Wei et al. (1999)
E1784K	12-year-old boy with multiple syncopes, a slow heart rate (42 bpm) and a prolonged QT interval (QTc of 509 ms)	Na^+ channels expressed in tsA201 cells reveal a +9-mV shift in voltage of half-activation, a −14-mV shift in voltage of half-inactivation, a persistent current of ≈1.5% of peak I_{Na} at −30 mV, and a faster recovery from inactivation	Deschênes et al. (2000)
E1784K	41 (heterozygous) mutation carriers from 15 families showed a prolonged QTc (mean ± SD of 485 ± 30 ms versus 402 ± 31 ms in the non-carriers) with a penetrance of 93%; sinus node dysfunction was present in 16 of the mutation carriers and 4 of these 16 also exhibited Brugada syndrome	Na^+ channels expressed in tsA201 cells reveal an ≈40% decrease in peak I_{Na}, a +12.5-mV shift in the voltage of half-activation, a −15.0-mV shift in the voltage of half-inactivation, a 3.5-fold larger persistent I_{Na} than that of wild-type, and an enhanced slow inactivation in absence of changes in recovery from inactivation	Makita et al. (2008)

(Continued)

Mutation	Clinical observations	Functional effects	Source
1795insD	ECG features of both LQTS and Brugada syndrome in the same mutation carriers from a large family; sinus bradycardia; sudden death at rest	Na^+ channels expressed in *Xenopus* oocytes reveal a +8.1-mV shift in steady-state activation curve, a −7.3-mV shift in steady-state inactivation curve, and a 78% decrease in peak current amplitude	Bezzina et al. (1999)
1795insD	Overlap syndrome in a large family; mutation carriers showing ECG features of long-QT syndrome, Brugada syndrome, conduction disease, and/or sinus bradycardia	—	Van den Berg et al. (2001); Van den Berg et al. (2002); Postema et al. (2009)
1795insD	—	1795insD channels expressed in HEK-293 cells reveal a persistent current of ≈1.4% of peak current amplitude at −20 mV, as compared to effectively zero for wild-type	Veldkamp et al. (2000); Veldkamp et al. (2003)
1795insD	Bradycardia, QT prolongation and right ventricular conduction slowing in heterozygous mice carrying the *Scn5a*-1798insD mutation (equivalent to human 1795insD)	A 39% decreased peak I_{Na} and a doubled persistent current in isolated heterozygous ventricular myocytes compared to wild-type ventricular myocytes	Remme et al. (2006)

More data on E1784K are available, e.g. in studies by Takahashi et al. (2014) and Veltmann et al. (2016).

Table S4. Mutations in *ANK2* underlying both sinus bradycardia and LQTS in Ankyrin-B Syndrome ('LQT4')

Mutation	Clinical observations	Functional effects	Source
E1425G	Severe sinus bradycardia and QTc prolongation in a four-generation family	—	Schott et al. (1995)
E1425G	Sinus bradycardia and QTc prolongation in 20 out of 23 genotype-positive family members; sinus bradycardia, QTc prolongation, and sudden cardiac death in *AnkB*$^{+/-}$ mice	Reduced levels of Na/K pump, Na/Ca exchanger, inositol-1,4,5-trisphosphate receptors ($InsP_3R$), and elevated peak $[Ca^{2+}]_i$ in isolated murine myocytes	Mohler et al. (2003)
E1425G	Sinus bradycardia in a large family (56 ± 15 bpm in mutation carriers vs. 85 ± 24 bpm in non-carriers); prolonged QTU interval	Both I_{NCX} (≈50%) and $I_{Ca,L}$ (>60%) reduced in SAN cells of *AnkB*$^{+/-}$ mice; no significant difference in $I_{Ca,T}$ and I_f; expression of Na/Ca exchanger and Na/K pump reduced by 30–40% on immunoblots	Le Scouarnec et al. (2008)
R1788W	Mild sinus bradycardia and QTc prolongation in two unrelated adult females	R1788W does not rescue abnormal expression or localization of Na/K pump, Na/Ca exchanger, or $InsP_3Rs$ in ankyrin-B$^{+/-}$ neonatal mouse cardiomyocytes	Mohler et al. (2004)
I1855R	Sinus bradycardia and QTc prolongation in a 49-year-old man; bradycardia in his mutation carrying sister	—	Ichikawa et al. (2016b)

Table S5. Mutations in *KCNE1* underlying both sinus bradycardia and LQTS type 5 (LQT5)

Mutation	Clinical observations	Functional effects	Source
A8V	Prominent sinus bradycardia (46 bpm) and marked QT prolongation (QTc 600 ms) in a 63-year-old female patient	No effect of A8V mutant compared to wild-type *KCNE1* if co-expressed with wild-type *KCNQ1* in COS-7 cells; >40% reduction in I_{Kr} density compared to wild-type *KCNE1* if co-expressed with wild-type *KCNH2* in CHO cells	Ohno et al. (2007)
D85N	Three adult females with sinus bradycardia and QTc prolongation among 24 carriers of the D85N mutation	Homozygous co-expression of the mutant with wild-type *KCNQ1* or *KCNH2* in CHO cells reveals significant loss-of-function effects on both I_{Ks} and I_{Kr}	Nishio et al. (2009)
D85N	Mild bradycardia and QTc interval of 470 ms in an 11-year-old female competitive athlete	Homozygous co-expression of the mutant with *KCNH2* in tsA201 cells reduced I_{Kr} tail current by 85%, whereas heterozygous co-expression reduced the current by 52%	Nof et al. (2011)
D85N	Sinus bradycardia and QTc prolongation (515 ms) in a 20-year-old female (46 bpm) and her younger sister (47 bpm; 532 ms)	—	Nakajima et al. (2010)

(Continued)

**Mutation**	**Clinical observations**	**Functional effects**	**Source**
R98W	Sinus bradycardia (45 bpm) with mild QT prolongation (QTc 460 ms) in a 19-year-old female	≈50% reduction in I_{Ks} density and +16-mV shift in voltage of half-activation compared to wild-type *KCNE1* if co-expressed with wild-type *KCNQ1* in COS-7 cells; no effect of R98W mutant compared to wild-type *KCNE1* if co-expressed with wild-type *KCNH2* in CHO cells	Ohno et al. (2007)

D85N also observed in a carrier of the S338F mutation in *KCNQ1* exhibiting sinus bradycardia and profound QTc prolongation (cf. Table S1).

Table S6. Mutations in *KCNE2* underlying both sinus bradycardia and LQTS type 6 (LQT6)

Mutation	Clinical observations	Functional effects	Source
M54T	Sinus bradycardia (39 bpm) and QTc prolongation (476 ms) in a 51-year-old male	Co-expression of HCN4 and M54T mutant MiRP1 in cultured newborn rat ventricular myocytes reduces HCN4 current (I_f) amplitude by ≈80% and slows activation by a factor of 2 in comparison to co-expression of HCN4 and wild-type MiRP1	Nawathe et al. (2013)
M54T	38-year-old female who had VF while jogging; atypical response to exercise with QTc intervals ranging from 390 to 500 ms	Co-expression of *KCNH2*-encoded HERG α-subunits and M54T mutant MiRP1 in CHO cells forms I_{Kr} channels that deactivate twice as fast as upon co-expression with wild-type MiRP1	Abbott et al. (1999)
M54T	—	Co-expression of *KCNH2*-encoded HERG α-subunits and M54T mutant MiRP1 in CHO cells reveals a 39% decrease in current density as compared to co-expression with wild-type MiRP1	Sesti et al. (2000)
M54T	—	+10-mV shift in voltage of half-activation and −10-mV shift in voltage of half-inactivation compared to wild-type MiRP1 if co-expressed with wild-type HERG in CHO cells; slowing of deactivation at physiological potentials	Lu et al. (2003)

(Continued)

Mutation	Clinical observations	Functional effects	Source
V65M	QTc prolongation, but not sinus bradycardia, observed in 17-year-old female; sinus bradycardia (55 bpm), but not QTc prolongation, observed in the genotype-positive father	CHO cells co-expressing wild-type HERG and wild-type or mutant MiRP1 reveal an accelerated I_{Kr} inactivation time course in case of mutant MiRP1	Isbrandt et al. (2002)

Table S7. Mutations in *CACNA1C* underlying both sinus bradycardia and LQTS type 8 (‘LQT8’)[a]

Mutation	Clinical observations	Functional effects	Source
A582D	12-year-old girl with significantly prolonged QTc interval and sinus bradycardia (50 bpm)	Expression of mutant channels in CHO cells reveals a slower course of inactivation of $I_{Ca,L}$ as compared to wild-type	Fukuyama et al. (2014)
P857R	Mild QT prolongation in 7 mutation carriers from one family; sinus bradycardia and a QTc of 454 ms in a male mutation carrier	Expression of mutant channels in HEK-293 cells results in a >100% increase in $I_{Ca,L}$ density as compared to wild-type; biotinylation experiments show a 64% increase in surface membrane expression of mutant *CACNA1C* relative to wild-type	Boczek et al. (2013)
R858H	15-year-old boy with a prolonged QT interval and severe bradycardia (44 bpm)	Expression of mutant channels in CHO cells reveals a significant increase in $I_{Ca,L}$ density as compared to wild-type	Fukuyama et al. (2014)

[a] Not taken into account are mutations associated with Timothy syndrome (G402S, G406R), which has been named LQT8, but above all is a multisystem disorder, including bradycardia and extreme QT prolongation (Splawski et al., 2004; Splawski et al., 2005). Also, bradycardia is caused by 2:1 atrioventricular block rather than sinus bradycardia. A table with clinical and genetic characteristics of Timothy syndrome patients with proven genetic diagnosis reported in the literature is provided by Sepp et al. (2017).

Table S8. Mutations underlying both sinus bradycardia and one of LQTS types 9–16 (LQT9–LQT16)[a]

LQTS type	Mutation	Clinical observations	Functional effects	Source
LQT9	*CAV3*-T78M[b]	Sinus bradycardia in two unrelated LQTS patients	—	Vatta et al. (2006)
LQT10	*SCN4B*-L179F	Asymptomatic bradycardia and profound QT prolongation in a 21-month-old girl	3-fold increase in late sodium current, +3-mV shift in voltage of half-inactivation, and slowed recovery from inactivation (as compared to wild-type Navβ4)	Medeiros-Domingo et al. (2007)
LQT14	*CALM1*-E105A	6-year-old male with sinus bradycardia and QT prolongation; de novo mutation	—	Takahashi et al. (2016)
LQT14	*CALM1*-F142L	14-year-old male with severe cognitive disability, epilepsy, markedly prolonged QTc, and nocturnal bradycardia	Impaired Ca^{2+} binding by mutant calmodulin C domains	Crotti et al. (2013)
LQT14	*CALM1*-F142L	Bradycardia and QT prolongation in neonate male; de novo mutation; sudden cardiac death at 1 year of age	—	Boczek et al. (2016)
LQT15	*CALM2*-D96V	Female infant with sinus bradycardia and markedly prolonged QTc (690 ms)	Impaired Ca^{2+} binding by mutant calmodulin C domains	Crotti et al. (2013)

(Continued)

LQTS type	Mutation	Clinical observations	Functional effects	Source
LQT15	*CALM2*-N98I	Sinus bradycardia and a prolonged QTc interval (555 ms) in 2-year-old boy	Impaired Ca^{2+} binding by mutant calmodulin C domains as determined by changes in intrinsic fluorescence in *Escherichia coli* expressing wild-type and mutant calmodulins	Makita et al. (2014)
LQT15	*CALM2*-D132H	Profound sinus bradycardia and prolonged QT interval (QTc 651 ms) in 1-day-old infant	Human induced pluripotent stem cell-derived cardiomyocytes show impaired calcium-dependent inactivation of the voltage-gated calcium current; no changes in voltage-dependent inactivation	Pipilas et al. (2016)
LQT16	*CALM3*-D96H	Sinus bradycardia and extreme QTc interval prolongation (627 ms) in 3-day-old girl	—	Chaix et al. (2016)
LQT16	*CALM3*-F142L	Sinus bradycardia and extreme QTc interval prolongation (645 ms) in 1-day-old girl	—	Chaix et al. (2016)

[a] Mutations in *AKAP9* (LQT11), encoding the anchoring protein yotiao, in *SNTA1* (LQT12), encoding the scaffolding protein α1-syntrophin, and in *KCNJ5* (LQT13), encoding the Kir3.4 (or GIRK4) subunit of the $I_{K,ACh}$ channel, decrease I_{Ks} and inhibit its functional response to cAMP (Chen et al., 2007), increase peak and late I_{Na} (Ueda et al., 2008; Wu et al., 2008), and reduce $I_{K,ACh}$, respectively. No data on simultaneous intrinsic sinus bradycardia have been reported.

[b] Mutations in *CAV3* may affect multiple ion currents, not only increasing late I_{Na} (Vatta et al., 2006) and decreasing I_{K1} (Vaidyanathan et al., 2013), but also affecting I_f (Ye et al., 2008; Campostrini et al., 2017) and the ultrarapid delayed rectifier K^+ current (I_{Kur}) (Campostrini et al., 2017).

2 Computer Simulations

To illustrate the action of the 1795insD mutation in *SCN5A*, we used data from computer simulations that we recently carried out during another study (Wilders, 2018). Effects of the 1795insD mutation on the fast sodium current (I_{Na}) were implemented in the CellML code (Cuellar et al., 2003) of the Fabbri-Severi computational model of a human SAN pacemaker cell (Fabbri et al., 2017). The CellML code was edited and run in version 0.9.31.1409 of the Windows based Cellular Open Resource (COR) environment (Garny et al., 2003). In some of the simulations, the control beating rate of the model cell of 74 bpm was lowered to 49 bpm (vagal activity) through the simulated administration of 20 nM acetylcholine (ACh), which acts through activation of the ACh-activated K^+ current ($I_{K,ACh}$) as well as inhibition of the hyperpolarization-activated 'funny current' (I_f), the L-type calcium current ($I_{Ca,L}$), and the sarco/endoplasmic reticulum Ca^{2+}-ATPase (SERCA pump), as detailed by Fabbri et al. (2017). All simulations were run for a simulated period of 200 s, which is a sufficiently long time to reach steady-state behavior. Data from the final 10 s are shown.

To simulate the 1795insD mutation in *SCN5A*, the model I_{Na} was split into a wild-type and a mutant component, thus representing the heterozygous nature of the mutation. The fully-activated conductance of the wild-type component was set to 50% of the control value. The mutant component differed from the wild-type component in several respects. First, the voltage dependence of the equations governing the I_{Na} activation and inactivation kinetics was shifted by +8.1 and −7.3 mV, respectively, to implement the experimentally observed +8.1 and −7.3 mV shifts in the steady-state activation and inactivation curves (Bezzina et al., 1999). Second, the fully-activated conductance was set to 25% of the control value, which was required to match the decrease in peak I_{Na} that was observed in voltage clamp experiments (Bezzina et al., 1999). Third, 0.6% of the mutant I_{Na} channels were made non-inactivating to introduce a late I_{Na} current with an amplitude of 1.0–1.5% of the peak I_{Na} recorded in voltage clamp mode, in accordance with the late I_{Na} that was observed experimentally (Veldkamp et al., 2000; Veldkamp et al., 2003).

(Continued on next page)

3 References

Abbott, G. W., Sesti, F., Splawski, I., Buck, M. E., Lehmann, M. H., Timothy, K. W., et al. (1999). MiRP1 forms I_{Kr} potassium channels with HERG and is associated with cardiac arrhythmia. *Cell* 97, 175–187. doi: 10.1016/S0092-8674(00)80728-X

Aidery, P., Kisselbach, J., Schweizer, P. A., Becker, R., Katus, H. A., and Thomas, D. (2012). Impaired ion channel function related to a common *KCNQ1* mutation — Implications for risk stratification in long QT syndrome 1. *Gene* 511, 26–33. doi: 10.1016/j.gene.2012.09.041

Bdier, A. Y., Al-Ghamdi, S., Verma, P. K., Dagriri, K., Alshehri, B., Jiman, O. A., et al. (2017). Autosomal recessive long QT syndrome, type 1 in eight families from Saudi Arabia. *Mol. Genet. Genomic Med.* 5, 592–601. doi: 10.1002/mgg3.305

Beery, T. A., Shooner, K. A., and Benson, D. W. (2007). Neonatal long QT syndrome due to a de novo dominant negative *hERG* mutation. *Am. J. Crit. Care.* 16, 412–416. doi: —

Bellocq, C., Wilders, R., Schott, J.-J., Louérat-Oriou, B., Boisseau, P., Le Marec, H., et al. (2004). A common antitussive drug, clobutinol, precipitates the long QT syndrome 2. *Mol. Pharmacol.* 266, 1093–1102. doi: 10.1124/mol.104.001065

Bennett, P. B., Yazawa, K., Makita, N., and George, A. L. Jr. (1995). Molecular mechanism for an inherited cardiac arrhythmia. *Nature* 376, 683–685. doi: 10.1038/376683a0

Bezzina, C., Veldkamp, M. W., Van den Berg, M. P., Postma, A. V., Rook, M. B., Viersma, J.-W., et al. (1999). A single Na^+ channel mutation causing both long-QT and Brugada syndromes. *Circ. Res.* 85, 1206–1213. doi: 10.1161/01.RES.85.12.1206

Bhuiyan, Z. A., Momenah, T. S., Amin, A. S., Al-Khadra, A. S., Alders, M., Wilde, A. A. M., et al. (2008). An intronic mutation leading to incomplete skipping of exon-2 in *KCNQ1* rescues hearing in Jervell and Lange-Nielsen syndrome. *Prog. Biophys. Mol. Biol.* 98, 319–327. doi: 10.1016/j.pbiomolbio.2008.10.004

Biliczki, P., Girmatsion, Z., Brandes, R. P., Harenkamp, S., Pitard, B., Charpentier, F., et al. (2009). Trafficking-deficient long QT syndrome mutation KCNQ1-T587M confers severe clinical phenotype by impairment of KCNH2 membrane localization: evidence for clinically significant I_{Kr}-I_{Ks} α-subunit interaction. *Heart Rhythm* 6, 1792–1801. doi: 10.1016/j.hrthm.2009.08.009

Boczek, N. J., Best, J. M., Tester, D. J., Giudicessi, J. R., Middha, S., Evans, J. M., et al. (2013). Exome sequencing and systems biology converge to identify novel mutations in the L-type calcium channel, *CACNA1C*, linked to autosomal dominant long QT syndrome. *Circ. Cardiovasc. Genet.* 6, 279–289. doi: 10.1161/CIRCGENETICS.113.000138

Boczek, N. J., Gomez-Hurtado, N., Ye, D., Calvert, M. L., Tester, D. J., Kryshtal, D. O., et al. (2016). Spectrum and prevalence of *CALM1*-, *CALM2*-, and *CALM3*-encoded calmodulin variants in long QT syndrome and functional characterization of a novel long QT syndrome-associated calmodulin missense variant, E141G. *Circ. Cardiovasc. Genet.* 9, 136–146. doi: 10.1161/CIRCGENETICS.115.001323

Brink, P. A., Crotti, L., Corfield, V., Goosen, A., Durrheim, G., Hedley, P., et al. (2005). Phenotypic variability and unusual clinical severity of congenital long-QT syndrome in a founder population. *Circulation* 112, 2602–2610. doi: 10.1161/CIRCULATIONAHA.105.572453

Burgess, D. E., Bartos, D. C., Reloj, A. R., Campbell, K. S., Johnson, J. N., Tester, D. J., et al. (2012). High-risk long QT syndrome mutations in the Kv7.1 (KCNQ1) pore disrupt the molecular basis for rapid K^+ permeation. *Biochemistry* 51, 9076–9085. doi: 10.1021/bi3009449

Campostrini, G., Bonzanni, M., Lissoni, A., Bazzini, C., Milanesi, R., Vezzoli E., et al. (2017). The expression of the rare caveolin-3 variant T78M alters cardiac ion channels function and membrane excitability. *Cardiovasc. Res.* 113, 1256–1265. doi: 10.1093/cvr/cvx122

Chaix, M.-A., Koopmann, T. T., Goyette, P., Alikashani, A., Latour, F., Fatah, M., et al. (2016). Novel *CALM3* mutations in pediatric long QT syndrome patients support a *CALM3*-specific calmodulinopathy. *HeartRhythm Case Rep.* 2, 250–254. doi: 10.1016/j.hrcr.2016.02.002

Chandra, R., Starmer, C. F., and Grant, A. O. (1998). Multiple effects of KPQ deletion mutation on gating of human cardiac Na^+ channels expressed in mammalian cells. *Am. J. Physiol.* 274, H1643–H1654. doi: 10.1152/ajpheart.1998.274.5.H1643

Chen, L., Marquardt, M. L., Tester, D. J., Sampson, K. J., Ackerman, M. J., and Kass, R. S. (2007). Mutation of an A-kinase-anchoring protein causes long-QT syndrome. *Proc. Natl. Acad. Sci. U. S. A.* 104, 20990–20995. doi: 10.1073/pnas.0710527105

Crotti, L., Johnson, C. N., Graf, E., De Ferrari, G. M., Cuneo, B. F., Ovadia, M., et al. (2013). Calmodulin mutations associated with recurrent cardiac arrest in infants. *Circulation* 127, 1009–1017. doi: 10.1161/CIRCULATIONAHA.112.001216

Cuellar, A. A., Lloyd, C. M., Nielsen, P. F., Bullivant, D. P., Nickerson, D. P., and Hunter, P. J. (2003). An overview of CellML 1.1, a biological model description language. *Simulation* 79, 740–747. doi: 10.1177/0037549703040939

Deschênes, I., Baroudi, G., Berthet, M., Barde, I., Chalvidan, T., Denjoy, I., et al. (2000). Electrophysiological characterization of *SCN5A* mutations causing long QT (E1784K) and Brugada (R1512W and R1432G) syndromes. *Cardiovasc. Res.* 46, 55–65. doi: 10.1016/S0008-6363(00)00006-7

Dumaine, R., Wang, Q., Keating, M. T., Hartmann, H. A., Schwartz, P. J., Brown, A. M., et al. (1996). Multiple mechanisms of Na^+ channel-linked long-QT syndrome. *Circ. Res.* 78, 916–924. doi: 10.1161/01.RES.78.5.916

Fabbri, A., Fantini, M., Wilders, R., and Severi, S. (2017). Computational analysis of the human sinus node action potential: model development and effects of mutations. *J. Physiol.* 595, 2365–2396. doi: 10.1113/JP273259

Ficker, E., Dennis, A. T., Obejero-Paz, C. A., Castaldo, P., Taglialatela, M., and Brown, A. M. (2000). Retention in the endoplasmic reticulum as a mechanism of dominant-negative current suppression in human long QT syndrome. *J. Mol. Cell. Cardiol.* 32, 2327–2337. doi: 10.1006/jmcc.2000.1263

Fukuyama, M., Wang, Q., Kato, K., Ohno, S., Ding, W.-G., Toyoda, F., et al. (2014). Long QT syndrome type 8: novel *CACNA1C* mutations causing QT prolongation and variant phenotypes. *Europace* 16, 1828–1837. doi: 10.1093/europace/euu063

Garny, A., Kohl, P., and Noble, D. (2003). Cellular open resource (COR): a public CellML based environment for modelling biological function. *Int. J. Bifurcat. Chaos* 13, 3579–3590. doi: 10.1142/S021812740300882X

Guerrier, K., Czosek, R. J., Spar, D. S., and Anderson, J. (2013). Long QT genetics manifesting as atrial fibrillation. *Heart Rhythm* 10, 1351–1353. doi: 10.1016/j.hrthm.2013.07.012

Hamada, H., Horigome, H., Asaka, M., Shigemitsu, S., Mitsui, T., Kubo, T., et al. (1999). Prenatal diagnosis of long QT syndrome using fetal magnetocardiography. *Prenat. Diagn.* 19, 677–680. doi: 10.1002/(SICI)1097-0223(199907)19:7<677::AID-PD597>3.0.CO;2-Z

Heijman, J., Spätjens, R. L. H. M. G., Seyen, S. R. M., Lentink, V., Kuijpers, H. J. H., Boulet, I. R., et al. (2012). Dominant-negative control of cAMP-dependent I_{Ks} upregulation in human long-QT syndrome type 1. *Circ. Res.* 110, 211–219. doi: 10.1161/CIRCRESAHA.111.249482

Henrion, U., Zumhagen, S., Steinke, K., Strutz-Seebohm, N., Stallmeyer, B., Lang, F., et al. (2012). Overlapping cardiac phenotype associated with a familial mutation in the voltage sensor of the KCNQ1 channel. *Cell. Physiol. Biochem.* 29, 809–818. doi: 10.1159/000178470

Hoosien, M., Ahearn, M. E., Myerburg, R. J., Pham, T. V., Miller, T. E., Smets, M. J., et al. (2013). Dysfunctional potassium channel subunit interaction as a novel mechanism of long QT syndrome. *Heart Rhythm* 10, 728–737. doi: 10.1016/j.hrthm.2012.12.033

Horigome, H., Nagashima, M., Sumitomo, N., Yoshinaga, M., Ushinohama, H., Iwamoto, M., et al. (2010). Clinical characteristics and genetic background of congenital long-QT syndrome diagnosed in fetal, neonatal, and infantile life: a nationwide questionnaire survey in Japan. *Circ. Arrhythm. Electrophysiol.* 3, 10–17. doi: 10.1161/CIRCEP.109.882159

Huang, H., Kuenze, G., Smith, J. A., Taylor, K. C., Duran, A. M., Hadziselimovic, A., et al. (2018). Mechanisms of KCNQ1 channel dysfunction in long QT syndrome involving voltage sensor domain mutations. *Sci. Adv.* 4, eaar2631. doi: 10.1126/sciadv.aar2631

Ichikawa, M., Ohno, S., Fujii, Y., Ozawa, J., Sonoda, K., Fukuyama, M., et al. (2016a). Multigenerational inheritance of long QT syndrome type 2 in a Japanese family. *Intern. Med.* 55, 259–262. doi: 10.2169/internalmedicine.55.6014

Ichikawa, M., Aiba, T., Ohno, S., Shigemizu, D., Ozawa, J., Sonoda, K., et al. (2016b). Phenotypic variability of *ANK2* mutations in patients with inherited primary arrhythmia syndromes. *Circ. J.* 80, 2435–2442. doi: 10.1253/circj.CJ-16-0486

Isbrandt, D., Friederich, P., Solth, A., Haverkamp, W., Ebneth, A., Borggrefe, M., et al. (2002). Identification and functional characterization of a novel *KCNE2* (MiRP1) mutation that alters HERG channel kinetics. *J. Mol. Med.* 80, 524–532. doi: 10.1007/s00109-002-0364-0

Kagan, A., Yu, Z., Fishman, G. I., and McDonald, T. V. (2000). The dominant negative LQT2 mutation A561V reduces wild-type HERG expression. *J. Biol. Chem.* 275, 11241–11248. doi: 10.1074/jbc.275.15.11241

Keller, D. I., Acharfi, S., Delacrétaz, E., Benammar, N., Rotter, M., Pfammatter, J.-P., et al. (2003). A novel mutation in *SCN5A*, delQKP 1507–1509, causing long QT syndrome: role of Q1507 residue in sodium channel inactivation. *J. Mol. Cell. Cardiol.* 35, 1513–1521. doi: 10.1016/j.yjmcc.2003.08.007

Kinoshita, K., Komatsu, T., Nishide, K., Hata, Y., Hisajima, N., Takahashi, H., et al. (2014). A590T mutation in KCNQ1 C-terminal helix D decreases I_{Ks} channel trafficking and function but not Yotiao interaction. *J. Mol. Cell. Cardiol.* 72, 273–280. doi: 10.1016/j.yjmcc.2014.03.019

Le Scouarnec, S., Bhasin, N., Vieyres, C., Hund, T. J., Cunha, S. R., Koval, O., et al. (2008). Dysfunction in ankyrin-B-dependent ion channel and transporter targeting causes human sinus node disease. *Proc. Natl. Acad. Sci. U. S. A.* 105, 15617–15622. doi: 10.1073/pnas.0805500105

Li, G., Shi, R., Wu, J., Han, W., Zhang, A., Cheng, G., et al. (2016). Association of the hERG mutation with long-QT syndrome type 2, syncope and epilepsy. *Mol. Med. Rep.* 13, 2467–2475. doi: 10.3892/mmr.2016.4859

Lu, Y., Mahaut-Smith, M. P., Huang, C. L.-H., and Vandenberg, J. I. (2003). Mutant MiRP1 subunits modulate HERG K^+ channel gating: a mechanism for pro-arrhythmia in long QT syndrome type 6. *J. Physiol.* 551, 253–262. doi: 10.1111/j.1469-7793.2003.00253.x

Lupoglazoff, J.-M., Denjoy, I., Villain, E., Fressart, V., Simon, F., Bozio, A., et al. (2004). Long QT syndrome in neonates: conduction disorders associated with *HERG* mutations and sinus bradycardia with *KCNQ1* mutations. *J. Am. Coll. Cardiol.* 43, 826–830. doi: 10.1016/j.jacc.2003.09.049

Makita, N., Behr, E., Shimizu, W., Horie, M., Sunami, A., Crotti, L., et al. (2008). The E1784K mutation in *SCN5A* is associated with mixed clinical phenotype of type 3 long QT syndrome. *J. Clin. Invest.* 118, 2219–2229. doi: 10.1172/JCI34057

Makita, N., Yagihara, N., Crotti, L., Johnson, C. N., Beckmann, B.-M., Roh, M. S., et al. (2014). Novel calmodulin mutations associated with congenital arrhythmia susceptibility. *Circ. Cardiovasc. Genet.* 7, 466–474. doi: 10.1161/CIRCGENETICS.113.000459

Medeiros-Domingo, A., Kaku, T., Tester, D. J., Iturralde-Torres, P., Itty, A., Ye, B., et al. (2007). *SCN4B*-encoded sodium channel β4 subunit in congenital long-QT syndrome. *Circulation* 116, 134–142. doi: 10.1161/CIRCULATIONAHA.106.659086

Mehta, A., Sequiera, G. L., Ramachandra, C. J. A., Sudibyo, Y., Chung, Y., Sheng, J., et al. (2014). Re-trafficking of hERG reverses long QT syndrome 2 phenotype in human iPS-derived cardiomyocytes. *Cardiovasc. Res.* 102, 497–506. doi: 10.1093/cvr/cvu060

Mohler, P. J., Schott, J.-J., Gramolini, A. O., Dilly, K. W., Guatimosim, S., duBell, W. H., et al. (2003). Ankyrin-B mutation causes type 4 long-QT cardiac arrhythmia and sudden cardiac death. *Nature* 421, 634–639. doi: 10.1038/nature01335

Mohler, P. J., Splawski, I., Napolitano, C., Bottelli, G., Sharpe, L., Timothy, K., et al. (2004). A cardiac arrhythmia syndrome caused by loss of ankyrin-B function. *Proc. Natl. Acad. Sci. U. S. A.* 101, 9137–9142. doi: 10.1073/pnas.0402546101

Moss, A. J., Zareba, W., Benhorin, J., Locati, E. H., Hall, W. J., Robinson, J. L., et al. (1995). ECG T-wave patterns in genetically distinct forms of the hereditary long QT syndrome. *Circulation* 92, 2929–2934. doi: 10.1161/01.CIR.92.10.2929

Moss, A. J., Windle, J. R., Hall, W. J., Zareba, W., Robinson, J. L., McNitt, S., et al. (2005). Safety and efficacy of flecainide in subjects with long QT-3 syndrome (ΔKPQ mutation): a randomized, double-blind, placebo-controlled clinical trial. *Ann. Noninvasive Electrocardiol.* 10, 59–66. doi: 10.1111/j.1542-474X.2005.00077.x

Nakajima, T., Furukawa, T., Hirano, Y., Tanaka, T., Sakurada, H., Takahashi, T., et al. (1999). Voltage-shift of the current activation in *HERG* S4 mutation (R534C) in LQT2. *Cardiovasc. Res.* 44, 283–293. doi: 10.1016/S0008-6363(99)00195-9

Nakajima, T., Kaneko, Y., Manita, M., Iso, T., and Kurabayashi, M. (2010). Aborted cardiac arrest in a patient carrying KCNE1 D85N variant during the postpartum period. *Intern. Med.* 49, 1875–1878, doi: 10.2169/internalmedicine.49.3859

Nawathe, P. A., Kryukova, Y., Oren, R. V., Milanesi, R., Clancy, C. E., Lu, J. T., et al. (2013). An LQTS6 MiRP1 mutation suppresses pacemaker current and is associated with sinus bradycardia. *J. Cardiovasc. Electrophysiol.* 24, 1021–1027. doi: 10.1111/jce.12163

Nishio, Y., Makiyama, T., Itoh, H., Sakaguchi, T., Ohno, S., Gong, Y.-Z., et al. (2009). D85N, a KCNE1 polymorphism, is a disease-causing gene variant in long QT syndrome. *J. Am. Coll. Cardiol.* 54, 812–819. doi: 10.1016/j.jacc.2009.06.005

Nof, E., Barajas-Martinez, H., Eldar, M., Urrutia, J., Caceres, G., Rosenfeld, G., et al. (2011). LQT5 masquerading as LQT2: a dominant negative effect of *KCNE1*-D85N rare polymorphism on *KCNH2* current. *Europace* 13, 1478–1483. doi: 10.1093/europace/eur184

Nuyens, D., Stengl, M., Dugarmaa, S., Rossenbacker, T., Compernolle, V., Rudy, Y., et al. (2001). Abrupt rate accelerations or premature beats cause life-threatening arrhythmias in mice with long-QT3 syndrome. *Nat. Med.* 7, 1021–1027. doi: 10.1038/nm0901-1021

Ohno, S., Zankov, D. P., Yoshida, H., Tsuji, K., Makiyama, T., Itoh, H., et al. (2007). N- and C-terminal *KCNE1* mutations cause distinct phenotypes of long QT syndrome. *Heart Rhythm* 4, 332–340. doi: 10.1016/j.hrthm.2006.11.004

Omichi, C., Momose, Y., and Kitahara, S. (2010). Congenital long QT syndrome presenting with a history of epilepsy: misdiagnosis or relationship between channelopathies of the heart and brain? *Epilepsia* 51, 289–292. doi: 10.1111/j.1528-1167.2009.02267.x

Piippo, K., Laitinen, P., Swan, H., Toivonen, L., Viitasalo, M., Pasternack, M., et al. (2000). Homozygosity for a HERG potassium channel mutation causes a severe form of long QT syndrome: identification of an apparent founder mutation in the Finns. *J. Am. Coll. Cardiol.* 35, 1919–1925. doi: 10.1016/S0735-1097(00)00636-7

Pipilas, D. C., Johnson, C. N., Webster, G., Schlaepfer, J., Fellmann, F., Sekarski, N., et al. (2016). Novel calmodulin mutations associated with congenital long QT syndrome affect calcium current in human cardiomyocytes. *Heart Rhythm* 13, 2012–2019. doi: 10.1016/j.hrthm.2016.06.038

Postema, P. G., Van den Berg, M. P., Van Tintelen, J. P., Van den Heuvel, F., Grundeken, M., Hofman, N., et al. (2009). Founder mutations in the Netherlands: *SCN5a* 1795insD, the first described arrhythmia overlap syndrome and one of the largest and best characterised families worldwide. *Neth. Heart J.* 17, 422–428. doi: 10.1007/bf03086296

Remme, C. A., Verkerk, A. O., Nuyens, D., Van Ginneken, A. C. G., Van Brunschot, S., Belterman, C. N. W., et al. (2006). Overlap syndrome of cardiac sodium channel disease in mice carrying the equivalent mutation of human *SCN5A*-1795insD. *Circulation* 114, 2584–2594. doi: 10.1161/CIRCULATIONAHA.106.653949

Schott, J.-J., Charpentier, F., Peltier, S., Foley, P., Drouin, E., Bouhour, J.-B., et al. (1995). Mapping of a gene for long QT syndrome to chromosome 4q25-27. *Am. J. Hum. Genet.* 57, 1114–1122. doi: —

Schwartz, P. J., Spazzolini, C., Crotti, L., Bathen, J., Amlie, J. P., Timothy, K., et al. (2006). The Jervell and Lange-Nielsen syndrome: natural history, molecular basis, and clinical outcome. *Circulation* 113, 783–790. doi: 10.1161/CIRCULATIONAHA.105.592899

Sepp, R., Hategan, L., Bácsi, A., Cseklye, J., Környei, L., Borbás, J., et al. (2017). Timothy syndrome 1 genotype without syndactyly and major extracardiac manifestations. *Am. J. Med. Genet. A* 173, 784–789. doi: 10.1002/ajmg.a.38084

Sesti, F., Abbott, G. W., Wei, J., Murray, K. T., Saksena, S., Schwartz, P. J., et al. (2000). A common polymorphism associated with antibiotic-induced cardiac arrhythmia. *Proc. Natl. Acad. Sci. U. S. A.* 97, 10613–10618. doi: 10.1073/pnas.180223197

Siebrands, C. C., Binder, S., Eckhoff, U., Schmitt, N., and Friederich, P. (2006). Long QT 1 mutation KCNQ1^{A344V} increases local anesthetic sensitivity of the slowly activating delayed rectifier potassium current. *Anesthesiology* 105, 511–520. doi: 10.1097/00000542-200609000-00015

Spencer, C. I. (2009). Actions of ATX-II and other gating-modifiers on Na^+ currents in HEK-293 cells expressing WT and ΔKPQ hNav 1.5 Na^+ channels. *Toxicon* 53, 78–89. doi: 10.1016/j.toxicon.2008.10.015

Splawski, I., Timothy, K. W., Sharpe, L. M., Decher, N., Kumar, P., Bloise, R., et al. (2004). Cav1.2 calcium channel dysfunction causes a multisystem disorder including arrhythmia and autism. *Cell* 119, 19–31. doi: 10.1016/j.cell.2004.09.011

Splawski, I., Timothy, K. W., Decher, N., Kumar, P., Sachse, F. B., Beggs, A. H., et al. (2005). Severe arrhythmia disorder caused by cardiac L-type calcium channel mutations. *Proc. Natl. Acad. Sci. U. S. A.* 102, 8089–8096. doi: 10.1073/pnas.0502506102

Takahashi, K., Shimizu, W., Miyake, A., Nabeshima, T., Nakayashiro, M., and Ganaha H. (2014). High prevalence of the *SCN5A* E1784K mutation in school children with long QT syndrome living on the Okinawa islands. *Circ. J.* 78, 1974–1979. doi: 10.1253/circj.CJ-13-1516

Takahashi, K., Ishikawa, T., Makita, N., Takefuta, K., Nabeshima, T., and Nakayashiro, M. (2016). A novel de novo calmodulin mutation in a 6-year-old boy who experienced an aborted cardiac arrest. *HeartRhythm Case Rep.* 3, 69–72. doi: 10.1016/j.hrcr.2016.09.004

Thomas, D., Wimmer, A. B., Karle, C. A., Licka, M., Alter, M., Khalil, M., et al. (2005). Dominant-negative I_{Ks} suppression by KCNQ1-ΔF339 potassium channels linked to Romano-Ward syndrome. *Cardiovasc. Res.* 67, 487–497. doi: 10.1016/j.cardiores.2005.05.003

Ueda, K., Valdivia, C., Medeiros-Domingo, A., Tester, D. J., Vatta, M., Farrugia, G., et al. (2008). Syntrophin mutation associated with long QT syndrome through activation of the nNOS-SCN5A macromolecular complex. *Proc. Natl. Acad. Sci. U. S. A.* 105, 9355–9360. doi: 10.1073/pnas.0801294105

Vaidyanathan, R., Vega, A. L., Song, C., Zhou, Q., Tan, B.-H., Berger, S., et al. (2013). The interaction of caveolin 3 protein with the potassium inward rectifier channel Kir2.1: physiology and pathology related to long QT syndrome 9 (LQT9). *J. Biol. Chem.* 288, 17472–17480. doi: 10.1074/jbc.M112.435370

Van den Berg, M. P., Wilde, A. A. M., Viersma, J. W., Brouwer, J., Haaksma, J., Van der Hout, A. H., et al. (2001). Possible bradycardic mode of death and successful pacemaker treatment in a large family with features of long QT syndrome type 3 and Brugada syndrome. *J. Cardiovasc. Electrophysiol.* 12, 630–636. doi: 10.1046/j.1540-8167.2001.00630.x

Van den Berg, M. P., Viersma, J. W., Beaufort-Krol, G. C. M., Bink-Boelkens, M. T. E., Bezzina, C. R., Veldkamp, M. W., et al. (2002). A large family characterised by nocturnal sudden death. *Neth. Heart J.* 10, 304–312. doi: —

Vanoye, G. G., Desai, R. R., Fabre, K. L., Potet, F., DeKeyser, J.-M., Macaya, D., et al. (2017). High throughput functional evaluation of KCNQ1 decrypts variants of unknown significance. *bioRχiv* (preprint). doi: 10.1101/223206

Vatta, M., Ackerman, M. J., Ye, B., Makielski, J. C., Ughanze, E. E., Taylor, E. W., et al. (2006). Mutant caveolin-3 induces persistent late sodium current and is associated with long-QT syndrome. *Circulation* 114, 2104–2112. doi: 10.1161/CIRCULATIONAHA.106.635268

Veldkamp, M. W., Viswanathan, P. C., Bezzina, C., Baartscheer, A., Wilde, A. A. M., and Balser, J. R. (2000). Two distinct congenital arrhythmias evoked by a multidysfunctional Na^+ channel. *Circ. Res.* 86, e91–e97. doi: 10.1161/01.RES.86.9.e91

Veldkamp, M. W., Wilders, R., Baartscheer, A., Zegers, J. G., Bezzina, C. R., and Wilde, A. A. M. (2003). Contribution of sodium channel mutations to bradycardia and sinus node dysfunction in LQT3 families. *Circ. Res.* 92, 976–983. doi: 10.1161/01.RES.0000069689.09869.A8

Veltmann, C., Barajas-Martinez, H., Wolpert, C., Borggrefe, M., Schimpf, R., Pfeiffer, R., et al. (2016). Further insights in the most common *SCN5A* mutation causing overlapping phenotype of long QT syndrome, Brugada syndrome, and conduction defect. *J. Am. Heart Assoc.* 5, e003379. doi: 10.1161/JAHA.116.003379

Wang, Q., Shen, J., Splawski, I., Atkinson, D., Li, Z., Robinson, J. L., et al. (1995). *SCN5A* mutations associated with an inherited cardiac arrhythmia, long QT syndrome. *Cell* 80, 805–811. doi: 10.1016/0092-8674(95)90359-3

Wang, D. W., Yazawa, K., George, A. L. Jr., and Bennett, P. B. (1996). Characterization of human cardiac Na^+ channel mutations in the congenital long QT syndrome. *Proc. Natl. Acad. Sci. U. S. A.* 93, 13200–13205. doi: 10.1073/pnas.93.23.13200

Wang, Y., Shen, T., Fang, P., Zhou, J., Lou, K., Cen, Z., et al. (2018). The role and mechanism of chaperones Calnexin/Calreticulin in which ALLN selectively rescues the trafficking defective of HERG-A561V mutation. *Biosci. Rep.* BSR20171269. doi: 10.1042/BSR20171269

Wei, J., Wang, D. W., Alings, M., Fish, F., Wathen, M., Roden, D. M., et al. (1999). Congenital long-QT syndrome caused by a novel mutation in a conserved acidic domain of the cardiac Na^+ channel. *Circulation* 99, 3165–3171. doi: 10.1161/01.CIR.99.24.3165

Wilders, R. (2018). Sinus bradycardia in carriers of the *SCN5A*-1795insD mutation: unraveling the mechanism through computer simulations. *Int. J. Mol. Sci.* 19, 634. doi: 10.3390/ijms19020634

Wu, G., Ai, T., Kim, J. J., Mohapatra, B., Xi, Y., Li, Z., et al. (2008). α-1-syntrophin mutation and the long-QT syndrome: a disease of sodium channel disruption. *Circ. Arrhythm. Electrophysiol.* 1, 193–201. doi: 10.1161/CIRCEP.108.769224

Yamaguchi, M., Shimizu, M., Ino, H., Terai, H., Hayashi, K., Kaneda, T., et al. (2005). Compound heterozygosity for mutations Asp^{611}→Tyr in *KCNQ1* and Asp^{609}→Gly in *KCNH2* associated with severe long QT syndrome. *Clin. Sci.* 108, 143–150. doi: 10.1042/CS20040220

Yamashita, F., Horie, M., Kubota, T., Yoshida, H., Yumoto, Y., Kobori, A., et al. (2001). Characterization and subcellular localization of KCNQ1 with a heterozygous mutation in the C terminus. *J. Mol. Cell. Cardiol.* 33, 197–207. doi: 10.1006/jmcc.2000.1300

Ye, B., Balijepalli, R. C., Foell, J. D., Kroboth, S., Ye, Q., Luo, Y.-H., et al. (2008). Caveolin-3 associates with and affects the function of hyperpolarization-activated cyclic nucleotide-gated channel 4. *Biochemistry* 47, 12312–12318. doi: 10.1021/bi8009295

Zareba, W., Sattari, M. N., Rosero, S., Couderc, J. P., and Moss, A. J. (2001). Altered atrial, atrioventricular, and ventricular conduction in patients with the long QT syndrome caused by the ΔKPQ SCN5A sodium channel gene mutation. *Am. J. Cardiol.* 88, 1311–1314. doi: 10.1016/S0002-9149(01)02097-5
